# Increased Severity and Mortality in Adults Co-Infected with Malaria and HIV in Maputo, Mozambique: A Prospective Cross-Sectional Study

**DOI:** 10.1371/journal.pone.0088257

**Published:** 2014-02-05

**Authors:** Aase Berg, Sam Patel, Pål Aukrust, Catarina David, Miguel Gonca, Einar S. Berg, Ingvild Dalen, Nina Langeland

**Affiliations:** 1 Department of Medicine, Stavanger University Hospital, Stavanger, Norway; 2 Department of Medicine, The Central Hospital of Maputo, Maputo, Mozambique; 3 The Faculty of Medicine, The University of Bergen, Bergen, Norway; 4 Section of Clinical Immunology and Infectious Diseases, Oslo University Hospital Rikshospitalet, Oslo, Norway; 5 Department of Virology, The Norwegian Institute of Public Health, Oslo, Norway; 6 Department of Research, Stavanger University Hospital, Stavanger, Norway; Kenya Medical Research Institute - Wellcome Trust Research Programme, Kenya

## Abstract

**Background:**

Co-infection with falciparum malaria and HIV-1 increases the severity and mortality of both infections in unstable malaria-transmission areas. In contrast, in stable transmission areas, HIV co-infection increases the severity of both infections but has not been found to influence malaria mortality.

**Methods:**

In a prospective cross-sectional study, clinical and laboratory data were consecutively collected for all adults admitted with fever and/or suspected malaria to the medical department of the Central Hospital of Maputo, Mozambique, during two malaria seasons from January 2011. Malaria and HIV PCRs were performed, and risk factors for fatal outcomes were analysed. The impact of HIV on the clinical presentation and mortality of malaria was assessed.

**Findings:**

A total of 212 non-pregnant adults with fever and/or suspected malaria and 56 healthy controls were included in the study. Of the 131 patients with confirmed falciparum malaria, 70 were co-infected with HIV-1. The in-hospital mortality of the co-infected patients was 13.0% (9/69) compared with 1.7% (1/59) in the patients without HIV (p = 0.018). Malaria severity (p = 0.016) and co-infection with HIV (p = 0.064) were independent risk factors for death although the association with HIV did not reach statistical significance. The co-infected patients had significantly more frequent respiratory distress, bleeding disturbances, hypoglycaemia, liver and renal failure and high malaria parasitemia compared with the patients with malaria alone.

**Interpretations:**

HIV co-infection is associated with increased disease severity in and mortality from malaria in an area of stable malaria transmission. This finding was not observed earlier and should motivate doctors working in malaria-endemic areas to consider early HIV testing and a closer follow-up of patients with malaria and HIV co-infection.

## Introduction

Despite decreasing prevalence, malaria is one of the most important infectious diseases worldwide. In 2010, approximately 1.24 million people died of malaria globally, the majority of whom were in sub-Saharan Africa, including 52000 people in Mozambique. Of these individuals, 47% were above five years of age, which is more than previously assumed [1]. Malaria is meso-endemic in this country, with stable transmission of more than 95% due to *Plasmodium falciparum (P. falciparum)* [2], [3]. Mozambique has an estimated national human immunodeficiency virus type 1 (HIV1) prevalence in adults of 15–49 years of age of 11.5% in general and of 22.5% in Maputo (2009), with one of the highest global incidences of co-infection with malaria and HIV [4], [5], [6].

Several studies have suggested that people infected with HIV have more frequent and more severe episodes of malaria and *vice versa*, as parameters of HIV disease progression worsen in individuals during acute malaria episodes, with unknown long-term effects [7], [8], [9], [10], [11], [12]. While there is increased mortality in adults co-infected with malaria and HIV in areas with unstable malaria transmission, this phenomenon has not been established in areas with stable malaria transmission [13], [14], [15], [16].

In the present study, we examined the impact of co-infection with HIV on (i) clinical manifestations and (ii) outcome in hospitalised adult patients in Maputo with *P. falciparum* malaria.

## Methods

### Study design and participants

The Central Hospital of Maputo is a public quaternary care teaching hospital that serves Maputo's 1.2 million citizens and is a national referral hospital for the 22 million people in Mozambique. From 8^th^ January 2011 to 31^st^ March 2011 and from 7^th^ November 2011 to 14^th^ March 2012, a prospective cross-sectional study was performed on all patients consecutively admitted to the Medical Emergency Department at the Central Hospital in Maputo during workdays. All non-pregnant adults of 18 years or more and with an axillary temperature equal to or more than 38.0°C and/or suspected malaria were included, provided a given consent from the patient or from the next of kin if the patient was mentally confused or unconscious. “Suspected malaria” was defined as a history of fever, chills, headache, mental confusion, vomiting and/or diarrhoea, dyspnoea, myalgia and/or general malaise in the absence of symptoms, findings upon clinical examination or additional diagnostic tests indicating other infections. Additional diagnostic tests and exams were basic laboratory tests (e.g., Hemoglobin (Hb), WBC, differential count, ESR, AST, ALT, ALP, bilirubin, LDH, creatinine); other blood tests (e.g., bacteriological and fungal culture and antigen/antibodies for HIV-1, HIV-2, CMV, EBV, hepatitis B and C); urine analysis (stix, micro, culture); Cerebrospinal fluid (CSF) analysis (erythrocytes, WBC, differential count, protein, glucose, chloride, syphilis and cryptococcal tests, bacteriological and fungal culture); sputum analysis for *M. Tuberculosis* with microscopy (AAFB) and culture; stool analysis (e.g., microscopy for ova and cysts, bacteriological culture); and cytological/histological and different radiological exams, if indicated. In addition, health workers at the hospital and their family and friends were included as controls, provided that these individuals had a subjective feeling of wellbeing and a healthy appearance, as evaluated by the researchers. Female controls were excluded in the case of suspected or confirmed pregnancy.

### Procedures

A predefined set of clinical data was recorded from the patient files, which were compiled as a part of the routine clinical examination and follow-up at admission and during the hospital stay. Each file included the duration of symptoms, the presence of clinical criteria for severe malaria and HIV, the treatment given and the status at discharge (alive or dead) [17], [18]. If an inexperienced medical doctor had recorded the primary data, the researchers crosschecked these data. Fever was measured by a digital thermometer. (Bastos Medical, Valeo Corporation, Taipei, Taiwan.) Some of the patients who were both admitted and discharged directly from the Emergency Department had limited observations and laboratory findings. Survival data were crosschecked with the nurses' and the ward statistic manager's registries of deaths. For the healthy controls, only age and sex were recorded.

According to the hospital's routine, and consistent with standard procedures in the hospital's laboratory, we performed HIV testing (Determine, Alere Medical Co. Ltd; Chiba, Japan and Unigold, Trinity Biotech plc, Bray, Ireland), an HRP-2 Rapid Diagnostic Test (RDT) for malaria (2010–2011 First Response® Malaria antigen *P. falciparum*, Premium Medical Corporation Ltd., Daman, India; 2011–2012 ICT Malaria P.f.®, ICT Diagnostics Cape Town, South Africa), thick blood smears for malaria (Giemsa 20% for 5 minutes) and other routine laboratory tests. Parasitemia in a thick smear was categorised as +, which correlates to 1–10 parasites/100 fields; ++, or 11–100 parasites/100 fields; +++, or 1–10 parasites/field; ++++, or 11–100 parasites/field; or +++++, or >100 parasites/field [19]. In addition, blood samples were separately collected for HIV and malaria PCR analyses. The total nucleic acids were extracted from blood cell fractions [20]. Using 25% reduced sample volume; HIV-1 RNA was detected using an HIV-1 RG quantitative RT-PCR kit (Professional Biotech Pvt Ltd., New Delhi, India). Inhibitory samples were diluted 10 times and retested. Non-inhibitory, weakly positive samples were retested in a full-scale sample volume. Malaria PCR was performed using malaria plasmodium mitochondria- and species-specific 18S PCR. Divergent results were resolved by DNA sequencing [21]. An HCG urine pregnancy test was performed for female patients at fertile age with Quick Vue® (Quidel Corp., San Diego, California, USA).

Malaria positivity was defined as all patients with a positive malaria PCR test. Malaria PCR was not performed for two patients. These patients had positive HRP2 antigen tests and positive slides with parasitemia of 3+ or 5+ and were also defined as malaria positive. HIV positivity was defined as having a positive HIV serological test and/or a positive HIV PCR test. Malaria severity was categorised according to the number of criteria fulfilled for severe malaria, as defined by the WHO, and is given in Table 1 [22]. HIV disease severity was categorised according to the WHO's clinical HIV staging of I–IV [18].

**Table 1:** Malaria severity in relation to HIV status.

	Falciparum malaria		
Malaria severity criteria^1^	HIV+	HIV–	p^2^
n	70	61	
Hypotension, systolic BP<70	0 (0/63)	2 (1/52)	0.269
Respiratory distress, RR>30	25 (15/61)	6 (3/52)	0.006
Hyperpyrexia temp ≥40°C	6 (3/54)	12 (6/50)	0.243
GCS^3^<11 and/or convulsions	9 (6/70)	8 (5/61)	0.939
Bleeding disturbances and/or haemolysis	13 (9/70)	2 (1/61)	0.016
Jaundice and/or se-bilirubin >43 µmol/L	17 (13/70)	5 (3/61)	0.017
Hb<5 g/dL	15 (10/67)	5 (3/55)	0.092
Se-Glucose ≤2.2 mmol/L	8 (5/62)	0 (0/47)	0.046
Se-Creatinine >265 µmol/L	24 (15/63)	7 (3/46)	0.016
Malaria parasitemia of 4+ or 5+	52 (33/64)	30 (16/53)	0.020
Malaria severity score (mean and proportion of patients with severe malaria)^4^	1.46 (55/66)	0.44 (24/52)	<0.001

The data are percentage (the proportion of patients with given condition/the patients observed) unless otherwise indicated. Boldface type indicates statistical significance. To convert values for glucose levels to mg/dL, divide by 0.05551; to convert values for creatinine levels to mg/dL, divide by 88.4; and to convert values for bilirubin levels to mg/dL, divide by 17.1.

1 Malaria severity criteria modified from the malaria severity criteria given by the WHO.

2 The p-values are from Chi-squared tests (dichotomous data) and Mann-Whitney tests (malaria severity score).

3 Glascow Coma Scale.

4 Missing data 13 patients.

### Statistical analysis

**Figure 1:**
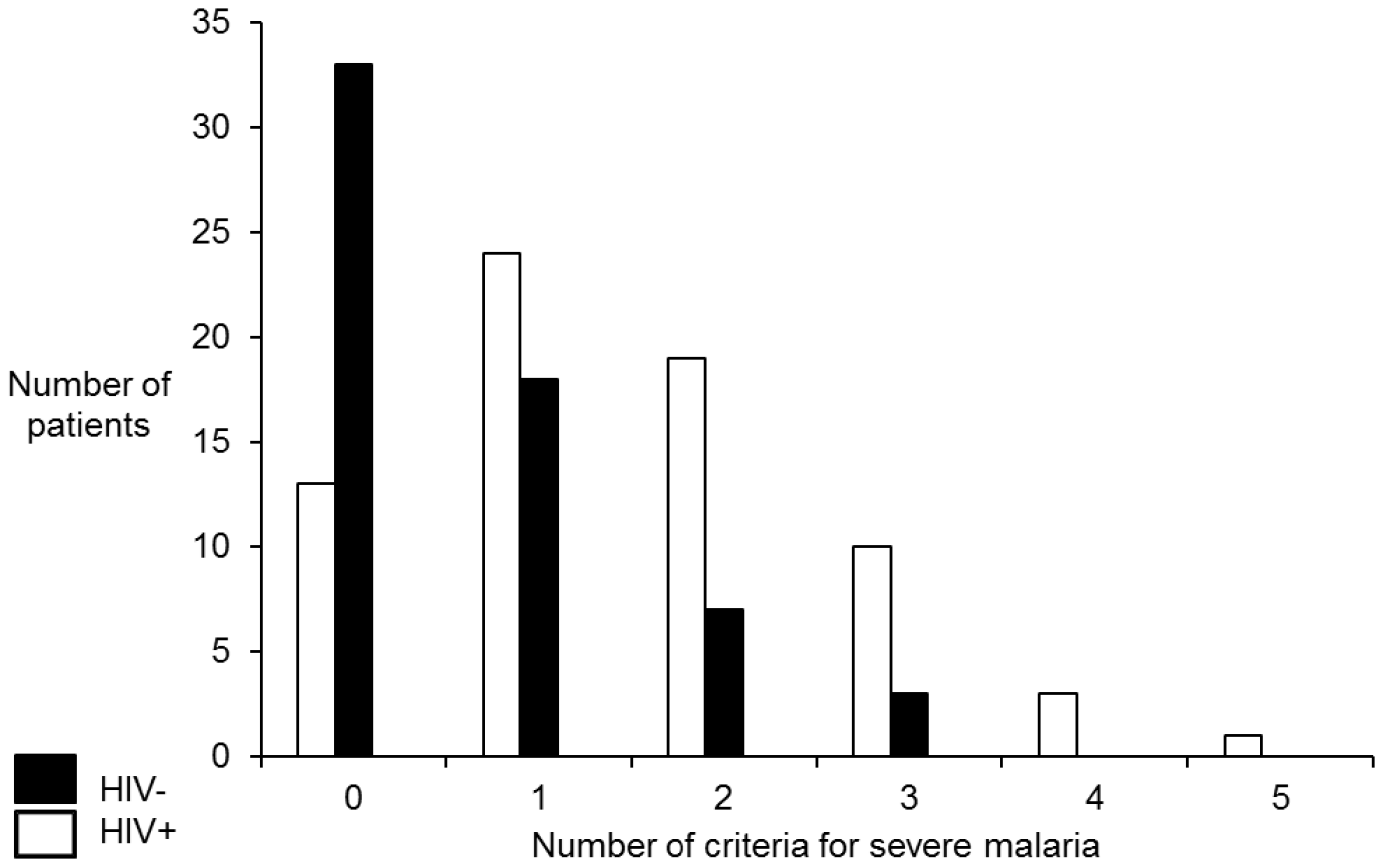
Malaria severity in patients with or without HIV-co-infection. Expressed in number of criteria fulfilled for severe malaria, as outlined in Table 2. (n = 118, missing 13 patients).

Categorical or dichotomous data were presented as counts and percentages, and comparisons between groups of malaria patients were performed using Chi-squared tests. Continuous data were presented as medians and ranges and compared using Mann-Whitney tests. The effects of possible risk factors for fatal outcome are evaluated in terms of odds ratios (ORs) with corresponding 95% confidence intervals (CIs) and p-values from Wald tests and simple and multiple binary logistic regression analyses. For analyses involving variables for which certain subjects/patients had missing observations, these subjects were excluded, and the number of included subjects was indicated in the result tables. When calculating number of fulfilment of the total ten severity criteria for malaria in table 1 and figure 1, patients were included even if missing few criteria done only on suspicion of actual organ involvement as bilirubin. All statistical analysis was performed with SPSS-21.

### Ethical consideration

Written consent or fingerprinting was obtained from patients or next of kin. The National Ethical Committee at the Ministry of Health in Mozambique and the Regional Ethical Committee in Eastern Norway approved the study. The institutional review board approved the use of health care workers as healthy controls.

## Results

### Characteristics of the study population

**Figure 2:**
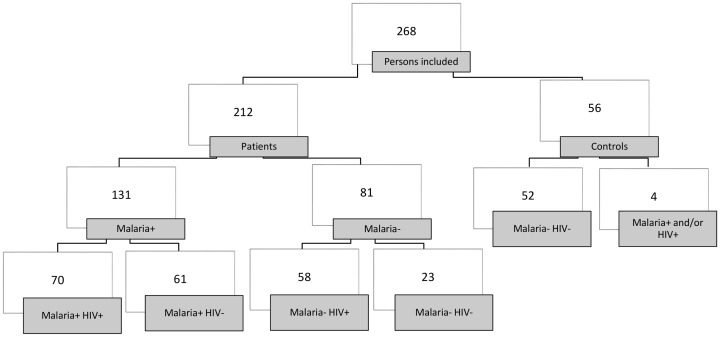
Flow diagram of the study population*. *Number of patients.

A total of 212 non-pregnant adults with fever and/or suspected malaria and 56 healthy controls were included in the study. In contrast, 48 eligible patients were excluded for different reasons (e.g., a lack of informed consent, leaving the hospital or not available within the hospital). In the entire patient group, the median age was 37 years (range: 18–84), 47% were women, and 99% were ethnic Mozambicans. Among the healthy controls, the median age was 26 years (range: 18–56), 41% were women, and 96% were ethnic Mozambicans. Most of the patients and most of the healthy controls came from the more peripheral suburbs of Maputo city. (Estimated ≥90%). Of the 212 patients with fever and/or suspected malaria, 131 (62%) had malaria and 70 of these patients (53%) were co-infected with HIV (Figure 2 and table 2). There was a significant difference in the HIV rate in malaria patients treated in and discharged from the Emergency Department compared with the admitted patients, with a rate of 29% (4/14) in those patients who were discharged and of 57% (60/106) in the patients who were admitted. (p = 0.048, missing data 10 patients). Of the healthy controls, four people were HIV positive, including one person who also had co-infection with *P. ovale*. All malaria patients were infected with *P. falciparum*. In addition, two patients had a double infection with *P. vivax* or *P. malariae*, respectively. All HIV-infected patients tested positive for HIV-1, except for one patient who had positive Determine and Unigold tests for HIV-2 but had an HIV PCR positive for HIV-1. Age and gender gave no significant difference between the malaria patients with and without HIV co-infection or between the HIV-positive patients with and without malaria. The median duration of symptoms in malaria patients with HIV was twice that of the malaria patients without HIV (8.6 vs.4.2 days) with a much wider range, although not significant. There was a significant difference in the median duration of symptoms for the HIV patients with and without malaria, from 8.6 days in the malaria- and HIV-infected patients to 7 days in the patients with HIV alone (p = 0.001). Of the malaria patients with HIV co-infection, 59% (41/70) had severe HIV infection, with HIV WHO stage 3 or 4, compared with 83% (48/58) of the HIV patients without malaria (p = 0.003). The median HIV viral load for the HIV patients with and without malaria was 1.8×10^4^ HIV RNA copies/mL plasma (range: 0 – 8.3×10^6^) (n = 61) and 1.3×10^4^ HIV RNA copies/mL plasma (range: 0–5.1×10^6^) (n = 58), respectively, without a significant difference (p = 0.184). Unfortunately, data on CD4 T cell counts were lacking in all except 11 and eight HIV patients with and without malaria, respectively, who had a median CD4 T cell count of 206 cells/µL (range: 14–632) and CD4 136 cells/µL (range: 10–196), respectively (p = 0.215). (Table 2)

**Table 2:** The characteristics of the study population^1^.

Characteristic	Malaria+ HIV+^2^	Malaria+ HIV-	Malaria-HIV+	p^3,4^	p^5^
n	70	61	58		
Case-fatality rate % (proportion)	13.0 (9/69)	1.7 (1/59)	29.1 (16/55)	0.017	0.030
Age in years, median (range)	40 (20–65)	40 (18–79)	38 (20–84)	0.487	0.233
Females, % (proportion)	50 (35/70)	39 (24/61)	50 (29/58)	0.223	1.0
Duration of symptoms in days, median (range)	8.6 (1–180)	4.2 (1–28)	7 (1–365)	0.191	0.001
Severe HIV^6^, % (proportion)	59 (41/70)	n.a.	83 (48/58)	n.a.	0.003
HIV viral load in copies/mL (median)	1.8×10^4^	n.a.	1.3×10^4^	n.a.	0.184
Median CD4 lymphocyte count in cells/µL^7^	206	n.a.	136	n.a.	0.215
Effective ART^8^ prior to admission % (proportion)	14 (9/64)	n.a.	19 (10/53)	n.a.	0.485

Boldface type indicates statistical significance.

1 98.5% of the study population were ethnic Mozambicans.

2 One patient with a positive malaria PCR test died of other causes than malaria, so his death is categorised as malaria negative.

3 The p-values are from Mann-Whitney tests (continuous data) or Chi-squared tests (dichotomous data).

4 Comparison of malaria patients with and without HIV co-infection (columns 1 and 2).

5 Comparison of HIV patients with and without malaria (columns 1 and 3).

6 Severe HIV  =  HIV WHO stage 3 or 4.

7 Please note the small numbers: n = 11 and n = 8.

8 ART  =  antiretroviral therapy  =  HIV treatment. “Effective” is defined as “Previous known ART and undetectable HIV-RNA in the plasma”. In relation to all HIV-patients with and without malaria.

### Clinical manifestations

Despite a history of fever, at admission, 50% (54/108) of the malaria patients presented with no fever, without a significant difference according to HIV status. The patients co-infected with HIV and malaria had significantly more frequent respiratory distress, bleeding disturbances and/or haemolysis, jaundice and/or elevated bilirubin levels, low serum glucose levels, renal failure and high malaria parasitemia compared with the patients with malaria alone. In line with this phenomenon, malaria patients with HIV co-infection had a significantly higher malaria severity score ( =  number of criteria for severe malaria) (p<0.001) (Table 1 and Figure 1). However, although those patients who were co-infected with HIV had an increased frequency of bleeding disturbance and/or haemolysis, there were no significant differences between the two groups in relation to platelet counts (<100×10^9^/L) or reduced haemoglobin levels (<5 g/dL). Moreover, there was no significant difference regarding the occurrence of cerebral confusion between the two groups (data not shown).

### Case-fatality rates

The in-hospital mortality rate of the patients with malaria and HIV was significantly higher than the mortality rate of the patients with malaria alone (13.0% (9/69) vs. 1.7% (1/59), p = 0.018), with missing data for two patients. In fact, of the 10 malaria patients who died, nine (90%) were HIV infected, and the tenth was 65 years of age. Patients admitted with fever of other causes than malaria had a HIV rate 72% (59/82) and a case fatality rate of 24% (19/78). Mortality was even higher for the patients with HIV alone (29.1% (16/55), p = 0.030) (Table 2). In total, 67% (77/115) had severe malaria, and 13% (10/77) died (p = 0.020), with missing data for 16 patients. Among the malaria patients, men had an increased risk of dying from malaria, with a crude odds ratio of 3.74 (0.76–18.35), but the difference from women in case-fatality rate was not significant (p = 0.086). The causes of death for the 10 malaria patients were cerebral malaria (three patients), complicated malaria with renal failure (three patients), coagulation disturbances (two patients), liver failure (one patient) and severe anaemia (one patient). Two of these patients had other severe comorbidity consisting of respectively longstanding pronounced alcohol abuse and lymphoma with recent chemotherapy. Although the co-morbidities could have contributed to the fatal outcome in these patients, these patients' histories, clinical findings, laboratory results and malaria parasitemia of 2+ (after quinine i.v. treatment for some days at the referring hospital) or 5+ suggested that malaria was the main cause of death. Moreover, exclusion of those two patients still resulted in significant increased mortality in the HIV-malaria patient group compared to the patients with malaria only (p = 0.042). A third patient had HIV and tuberculous meningoencephalitis according to history, clinical examination and laboratory investigation, but in addition, he had a *P. falciparum*-positive PCR result. This patient's cause of death was classified as meningoencephalitis and not malaria.

Malaria severity was the most important factor associated with increased in-hospital mortality. The patients who met three or more of the criteria for severe malaria had a crude odds ratio of 9.7 for death and a 95% CI of 2.43–38.91 (p<0.001) that persisted in multivariable analyses with an adjusted odds ratio of 6.3 and a 95% CI of 1.40–27.88 (p = 0.016) (Table 3 and Figure 1). The second most important factor associated with death was co-infection with HIV, with a crude odds ratio of 8.7 and a 95% CI of 8.7 (1.07–70.85) (p = 0.018). In multivariable analyses, the difference was not significant (p = 0.064), with an adjusted odds ratio of 8.0 and a 95% CI of 0.89–71.99.

**Table 3:** Risk factors for fatal outcome in malaria patients.

Characteristics		Case-fatality rate^1^ %	Crude OR^2^ (95% CI)	p^3^	Adjusted OR (95% CI^4^)	p^5^
Overall		7.8 (10/128)				
Age	18–49 years	6.3 (6/95)				
	50–79 years	12.1 (4/33)	1.26 (0.31–5.17)	0.752	2.3 (0.46–11.91)	p = 0.308
Sex	Women	3 (2/59)				
	Men	13.0 (9/69)	3.74 (0.76–18.35)	0.086	4.2 (0.76–3.40)	p = 0.100
HIV co-infection	No	1.7 (1/59)				
	Yes	13.0 (9/69)	8.7 (1.07–70.85)	0.018	8.0 (0.89–71.99)	p = 0.064
Malaria severity score	<3	4.5 (5/112)				
	≥3	31.3 (5/16)	9.7 (2.43–38.91)	<0.001	6.3 (1.40–27.88)	p = 0.016

Boldface type indicates statistical significance.

1 Deaths/number at risk in percentage and proportion.

2 OR  =  odds ratio.

3 The p-values are from Mann-Whitney tests.

4 CI  =  confidence interval.

5 The adjusted OR and p-values are from binary logistic regression.

### Treatment

Of the malaria patients, 92% received quinine intravenously as a first-line therapy, 4% received artemether intramuscularly, and the rest were treated with oral artemisinin combinations, without any significant differences in mortality. In total, 28% of the patients received antimicrobial therapy (ceftriaxone 27%, ampicillin 18%, penicillin 24% and other 31%). There was no statistically significant difference between the patients with and without HIV in receiving antimicrobials with a potential antimalarial effect, i.e., cotrimoxazole, ciprofloxacin, doxycycline and azithromycin [23], [24]. In total, 11% of the patients (14/131) received supplementary corticosteroid treatment, with no influence on outcome and with no difference between patients with and without HIV co-infection. At admission, 42% (54/128) of the HIV patients had a known HIV diagnosis. Of these patients, 56% (30/54) were on antiretroviral therapy (ART), of whom 63% (19/30) had undetectable HIV-RNA in the plasma (p = 0.001). The ARTs used were stavudine, lamivudine, efavirenz, zidovudine and/or nevirapine. Only eight patients reported receiving cotrimoxazole prophylaxis. There was no significant difference in the number of HIV-infected patients with and without malaria on the use of effective ART (i.e., with undetectable HIV-RNA) prior to admission, with respectively 69% (9/13) and 59% (10/17) (p = 0.558). Surprisingly, there was no significant difference in HIV severity or mortality in the patients with effective ART compared with those patients without ART. (Respectively p = 0.850 and p = 0.141). Of the HIV patients, 60% (44/74) were not receiving ART, despite an indication for ART*, due to a lack of HIV diagnosis before admission (*WHO HIV clinical stage 3 or 4). Of those patients with a known HIV diagnosis prior to admission, 86% (19/22) had an indication for ART but had failed to receive the HIV treatment.

## Discussion

In the present study, malaria patients co-infected with HIV had significantly more severe malaria compared with patients with malaria alone (Figure 1), which is consistent with several earlier studies [8], [9], [10], [11], [12]. Co-infection with HIV was significantly associated with increased in-hospital mortality. With more advanced immunodeficiency due to HIV, one would have expected increased mortality independent of the transmission area. This because primarily cellular immunity, but also humoral immunity, which are both important in malaria, are progressively affected by deterioration due to HIV disease [9]. However, several studies have failed to find increased mortality in endemic areas, which is in contrast to the findings of the present study [9], [13], [14], [15], [16]. These apparent discrepancies may have several explanations. First, in most of the previous studies, the sample size was relatively low (n<70) [9], [13], [14], [15], [16]. Second, the degree of HIV severity and the use of ART varied between the different studies [13], [15]. Third, in several of the previous studies, the ability to exclude relevant differential diagnoses was poor [9], [13]. Fourth, treatment for severe malaria varied between the different studies, with quinine used in most studies (including here) and artesunate used in other reports. This difference clearly could have influenced the outcome because artesunate is more effective than quinine [9], [13], [14], [15].

There was even higher mortality in the patients admitted with HIV without malaria, with 29.1% (16/55) compared with 13.2% (9/68) of the patients co-infected with HIV and malaria, which is most probably due to their advanced HIV-infection. (Table 2). The median duration of symptoms before admittance was 8.6, 4.2 and 7 days respectively in patients with malaria and HIV, malaria only and HIV only. HIV and malaria co-infected patients had a median duration of symptoms twice as long as HIV negative patients with malaria, although this was not statistically significant. Even if the median duration of symptoms between HIV-infected patients with and without malaria was rather modest, it reached statistical significance (Table 2). This again reflects the severity of disease in those two patient groups. The median viral load was about the same in the HIV-malaria group compared with the HIV group, despite less severe HIV disease. It is known that co-infection with malaria temporarily may boost HIV viremia [25]. There were also no significant differences in the median CD4 lymphocyte count or fraction on effective ART before admission, but the number of patients with known CD4 count was low (n = 8 and n = 11).

In comparison with the present investigation, a 2006 study on 51 patients with presumptive malaria, recruited at the same hospital as in the present study (Central Hospital of Maputo), had more severely ill patients, fewer patients on ART, a lower standard of care in the ward and a higher prevalence of both HIV and malaria. While there was no HIV-related increased malaria mortality, the diagnostic accuracy was poorer, the study group was smaller, and the treatment for severe malaria was artesunate rather than quinine [14]. Other studies also failed to find increased mortality, except for one retrospective study from Senegal on cerebral malaria [9], [15], [26]. However, the present study is, to the best of our knowledge, the first prospective study to show increased mortality in malaria patients co-infected with HIV in an area with stable malaria transmission. Importantly, the present study included patients with a wide spectrum of HIV-related immunodeficiency and had high diagnostic accuracy for both HIV and malaria.

Another possible reason for increased mortality in relation to co-infection with HIV in the present as opposed to the previous studies is that malaria transmission has decreased in Maputo, causing a gradual loss of malaria immunity [7]. This phenomenon would be expected to first be observed in immune-compromised patients, since it seems reasonable that an eventual reduction in the “average immunity” in the population will first be observed in patients with reduced immunity. However, most of the malaria patients in the current study came from the more peripheral parts of Maputo, rather than the city centre, where there is regular malaria exposure, and thus a lower risk of reduced immunity due to urban life. Whereas most literature has reported diminishing malaria prevalence in Mozambique, a recent WHO publication reported a steady increase in the number of confirmed malaria cases since 2009, but a reduction in hospital admissions and malaria-related deaths [27].

Another important point is whether positive malaria PCR may have been an incidental finding in several of the patients suffering from a different condition who happened to receive a malaria diagnosis after being subclinical carriers. In Manhiça in southern Mozambique, nearly half of the population was found to be carriers of *P. falciparum*, and most individuals were asymptomatic, with decreasing parasitemia with increasing age [28]. In contrast, in the Maputo area, there was a low prevalence of subclinical carriage, with 1.8% falciparum PCR positivity compared with 13.7% in the Mocuba District in Zambezia province [4]. Several studies have had similar findings, although asymptomatic falciparum carriage is particularly described in children, less in adolescents and usually even less in adults, but more in HIV positive persons [2], [4], [28], [29]. Asymptomatic carriage has likely not been a substantial problem in this case, since alternative diagnoses were not identified with a relatively effective diagnostic armamentarium. Second, only one of 56 healthy controls was a subclinical carrier (and then with *P. ovale* and not *P. falciparum*) in addition to being HIV positive. Third, the diagnosis at discharge agreed very well with the malaria PCR positivity (Cohen's kappa  =  0.88, data not shown).

Despite a history of fever, only 50% of the malaria PCR-positive malaria patients presented with a temperature of 38°C or more at admission, measured with the same reliable thermometer by the researchers. Could this be an indication, despite what was discussed above, that there is substantial subclinical carriage in this area, in certain subgroups differing from the healthy controls in this study. If so, we would have expected positive blood culture results in another parallel study of patients with fever. There is missing data for the use of paracetamol or other antipyretics, which may have influenced our findings. Alternatively, because the patients were admitted with a history of fever, these individuals may be in the stage of shifting from the cold to the warm phase or the opposite in the malaria cycle. If this was the case, similar studies may have lost patients if these studies used fever upon admittance as an inclusion criterion.

The only HIV-negative patient who died was 65 years of age, which is consistent with the known increased risk of malaria death with higher age in non-endemic areas [30]. In endemic areas, increased risk with age is not known, although there seems to be more frequent malaria in the age group above 50 years [31].

An important question in low-resource settings is whether there is a clinical entity of symptoms, signs and simple laboratory observations indicating that a patient is co-infected with HIV. In the current study, the co-infected patients had significantly more respiratory distress, bleeding disturbances, hypoglycaemia, liver and renal failure and high malaria parasitemia compared with patients with malaria alone. In several studies, the co-infected patients had more respiratory distress, renal failure, abdominal pain, diarrhoea or anaemia, whereas in other studies, there was no difference in clinical presentation [8], [13], [32]. The common denominator of these studies may well be that there is no specific clinical entity typical of co-infection with HIV and malaria; rather, there may be different indicators of disease severity, as also suggested by others [26].

The strength of the present study is its prospective nature, with the consecutive inclusion of a relatively large number of patients compared with similar studies and a high diagnostic accuracy for HIV and malaria. The main limitation is a possible selection bias of not including the poorest and the wealthiest patients because the wealthiest prefer private health care, and the poorest cannot pay the admission fee of approximately 5 USD if they do not have a referral letter. Because poverty is associated with an increased risk of malaria but a lower risk of HIV, this bias may have resulted in a potential loss of HIV-negative malaria patients, which could have influenced the observed mortality both ways [33]. The PCR method may have yielded false-positive malaria patients after treatment or false-negative HIV patients if these individuals were on ART without admitting its use during the hospital stay. The first mentioned issue could have given a too-low mortality rate, and the second issue could have yielded a too-high mortality rate. However, we assume that the second did not have a very important impact on the mortality data because there was supplementary HIV serology for most of the patients and a close follow-up in the ward. Two of the patients with severe malaria were not ethnic Mozambicans, but because both survived, this finding represents no bias. Lastly, even if most patients were severely ill, relatively few died, and the sample size may have been too low to detect other factors related to death.

## Conclusions

HIV co-infection is associated with increased mortality from malaria in an area of stable malaria transmission, in which both malaria severity and HIV are risk factors for death. The patients with HIV and malaria co-infection had significantly more frequent respiratory distress, bleeding disturbances, hypoglycaemia, liver and renal failure and high malaria parasitemia compared with the patients with malaria alone. Hence, early HIV testing should always be considered in patients with suspected malaria, and particularly with severe malaria, independent of the type of malaria transmission area. We hypothesise that this alertness of the physician may contribute to the increased survival of patients co-infected with HIV and malaria, due to awareness of potential complications at an early stage. As part of the search for improved therapy, future research is recommended to first confirm the present findings and to then elucidate the underlying immune mechanisms resulting in the observed HIV-associated increased malaria mortality.
